#  Varying mortality risks among patients with primary glomerular disease starting kidney replacement therapy: ERA Registry Figure of the month

**DOI:** 10.1093/ckj/sfaf237

**Published:** 2025-07-30

**Authors:** Vianda S Stel, Alberto Ortiz, Anneke Kramer

**Affiliations:** ERA Registry, Department of Medical Informatics, Amsterdam UMC–Location University of Amsterdam, Amsterdam, the Netherlands; Amsterdam Public Health Research Institute, Quality of Care, Amsterdam, the Netherlands; Department of Nephrology and Hypertension, IIS-Fundacion Jimenez Diaz UAM, Madrid, Spain; Department of Medicine, Universidad Autonoma de Madrid, Madrid, Spain; ERA Registry, Department of Medical Informatics, Amsterdam UMC–Location University of Amsterdam, Amsterdam, the Netherlands; Amsterdam Public Health Research Institute, Quality of Care, Amsterdam, the Netherlands

**Figure 1:**
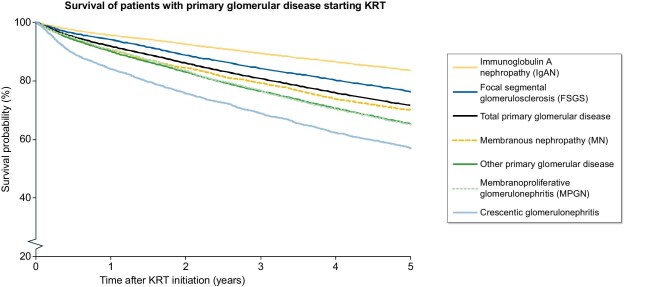
Five-year survival of patients starting kidney replacement therapy for kidney failure due to primary glomerular disease in Europe, by primary glomerular disease subgroup. **Explanation:** The crude 5-year patient survival of all patients who started kidney replacement therapy (KRT) between 2000 and 2014 for kidney failure due to primary glomerular disease (PGD) was 71.5%. Among PGD subgroups, the crude 5-year survival probability was lowest for crescentic glomerulonephritis (57.0%) and highest for immunoglobulin A nephropathy (83.6%). Nearly similar results were found after adjustment for age, sex, country and period of KRT initiation.

**Source:** Abd ElHafeez et al. NDT 2024, https://doi.org/10.1093/ndt/gfae034, Figure 3.

